# Audit Title: Diagnosis and Management of Mild Cognitive Impairment Within a Large Rural NHS Mental Health Trust

**DOI:** 10.1192/bjo.2026.11755

**Published:** 2026-06-30

**Authors:** Rachel Wright, Vlad Cucuiu

**Affiliations:** Lincolnshire Partnership Foundation Trust, Lincoln, United Kingdom

## Abstract

**Aims:**

The audit evaluated the diagnosis and management of mild cognitive impairment (MCI) across the older adult community mental health teams of a large and rural mental health trust. In the absence of specific NICE guidelines for MCI, the standards used in this audit were based on the current older adult (OA) memory assessment and management service (MAMS) protocol, the Manchester Consensus guideline recommendations and NICE guidance NG16 and NG97.

The aims of this audit were to assess compliance with guidelines, screening processes, diagnostic accuracy, patient and caregiver education, follow-up and monitoring as well as record keeping.

**Methods:**

This was a baseline retrospective audit of 80 patients who received a diagnosis of MCI from older adult community mental health teams (CMHTs) between January 2023 and January 2024. 10 patients were reviewed from each of the 8 CMHT teams. Electronic RiO records were reviewed for documentation. Patients with a diagnosis of MCI outside of this period were excluded.

**Results:**

Of the 80 patients reviewed, 13 were excluded. 67 patients (100%) were assessed using standardised cognitive assessment tools. 52 (78%) had functional impairment documented. 47 (70%) were screened for depression. Of those eligible, 10% had a documented medication review. 42% received information about lifestyle and risk reduction advice. 14 patients (21%) had cardiovascular risk reduction advice shared within GP letter. 67 (100%) were not prescribed antidementia medication. Forty-six patients (69%) received a follow-up review. Reasons why 13 patients (19%) were not followed up remain unaccounted for. 42 patients (91%) recalled had monitoring of cognitive function and daily living activities over time. Of the recalled, 35 patients (76%) had an updated IQCODE or detail of functioning impairment evidence in RiO notes. 14 patients (21%) had their diagnosis changed to dementia at the follow-up review. 11 patients (16%) were informed about and offered access to partaking in research.

**Conclusion:**

Compliance with the standards was variable. Strengths included compliance with using standardised cognitive assessment tools as well as avoidance of inappropriate prescribing of antidementia medications. There was also a high rate of reviews re-assessing cognition. Key gaps included medication reviews, depression screening, patient lifestyle and risk reduction advice, and research opportunities. Actions implemented included sharing audit findings across OA CMHTs and creation of a MAMS how-to guide for clinicians. The how-to guide includes a MAMS assessment checklist and a standard information pack for patients and carers. A follow-up audit is planned one year after the MAMS how-to guide is instated.

